# Personality Disorders and System Use Among Older Veterans Using the Veterans Health Administration

**DOI:** 10.1093/geroni/igaa057.1251

**Published:** 2020-12-16

**Authors:** Jenefer Jedele, Cameron Griffin, Sharon Nelson

**Affiliations:** Veterans Health Administration, Ann Arbor, Michigan, United States

## Abstract

Personality disorders (PD) are defined by longstanding patterns of dysfunctional behavior and associated with higher risk for adverse outcomes, such as suicide and comorbid mental health (MH). Despite recognition that PDs manifest differently as individuals age, few studies have examined PDs in older adults. VHA serves a significant population of older Veterans, providing a valuable opportunity to explore PD in older ages. Using FY2019 administrative data, we compare documented PDs, MH, and VHA use in a cohort of recent Veteran VHA users 50 years and older, across six age groups. At least one PD was documented for 0.8% of the total cohort, decreasing with age from 1.8% (50-54) to 0.2% (75+). Among those with PD, 98% had a comorbid MH disorder with the greatest percent having a major depressive disorder (69.7%). Except for dementia, comorbid mental health disorders generally decreased with age. Suicide risk flags were documented for 8.2% of cohort Veterans with PD compared to 0.7% with MH only. Behavior risk flags were documented for 4.3% of cohort Veterans with PD compared to 0.3% with MH only. The presence of both flags decreased with age. Compared to cohort Veterans with MH only, those with PD had more than twice the number of outpatient MH encounters and 1.5 times more psychiatric hospitalizations and ED visits for MH. Differences in care were consistent across age. However, differences in use were greatest for Veterans ages 55-64. The need to address crises resulting in the use of inpatient and ED is discussed.

